# Social media usage to share information in communication journals: An analysis of social media activity and article citations

**DOI:** 10.1371/journal.pone.0263725

**Published:** 2022-02-09

**Authors:** Yasemin Özkent

**Affiliations:** Department of Radio Television and Cinema, Selcuk University, Konya, Turkey; Universidade de Brasilia, BRAZIL

## Abstract

Social media has surrounded every area of life, and social media platforms have become indispensable for today’s communication. Many journals use social media actively to promote and disseminate new articles. Its use to share the articles contributes many benefits, such as reaching more people and spreading information faster. However, there is no consensus in the studies that to evaluate between tweeted and non-tweeted papers regarding their citation numbers. Therefore, it was aimed to show the effect of social media on the citations of articles in the top ten communication-based journals. For this purpose, this work evaluated original articles published in the top 10 communication journals in 2018. The top 10 communication-based journals were chosen based on SCImago Journal & Country Rank (cited in 2019). Afterward, it was recorded the traditional citation numbers (Google Scholar and Thompson-Reuters Web of Science) and social media exposure of the articles in January 2021 (nearly three years after the articles’ publication date). It was assumed that this period would allow the impact of the published articles (the citations and Twitter mentions) to be fully observed. Based on this assessment, a positive correlation between exposure to social media and article citations was observed in this study.

## Introduction

The social network has become a tool for bringing people together, allowing individuals to list the users they are connected to, and to see other users’ connections [1]. Social media platforms (blogs, social networking sites, microblogging, etc.) contain all Web 2.0-based services. Social media has surrounded every area of life, and social media platforms are indispensable for today’s communication [2]. Scientists from various fields frequently use SoMe, especially Twitter, in most of their professional activities [3].

Scientists often use social media platforms to produce and debate ideas, share real-time information, spread their research, and find collaborators [2, 4]. The way information is collected, disseminated and consumed has been significantly changed because social media by it is encompassing and easily accessible. There has been a significant increase in the number of studies related to social media with an increase in the use of the Internet [5]. The increased use of social media has also significantly affected how research is spread. Circulated articles through social media are more visible than not circulated articles [6]. Various scientific studies have examined this relationship, and most have found a positive correlation between article citations and Twitter exposure [7–9].

Studies focusing on new media technologies in connection with the digital age since the 2000s have taken an important place in communication studies [10]. In their extensive studies on research topics in communication journals, Elisabeth Günther and Emese Domahidi (2017) observed that the Internet and social media have become the most important focus for communication research, in parallel with classical media, such as TV or newspaper [10]. Social media is an important field of study and practice in both interdisciplinary and communication fields [11–14]. Social media research is encouraged in the field of communication, as people today present themselves through digitally networked platforms. Therefore, this study aimed to demonstrate the effect of social media on citation numbers of articles in communication-based journals. The relationship between the traditional citation numbers of articles and social media posts was analyzed in present study. Thus, it was aimed to shed light on the relationship between social media usage and the number of article citations in the field of communication.

### Literature review

The emergence of social media took place simultaneously with Web 2.0. With the introduction of Web 2.0 into our lives, the Internet has become individualized, and use of social networks increased gradually. The Internet has become an interactive virtual world from a read-only state, and it has brought a different dimension to communication [12]. Today, social media is a wide network of interactions where people from many areas [15]. In particular, Internet has become a part of life due to the widespread use of smartphones. The majority of people actively use social media in daily life [16]. A recent study has stated that 70% of peoples in the USA have at least one social media account nowadays. The peoples over 65 years old of 62% have a social media account and they are regularly on social media. This observation is also similar for adolescents. The using of social networks has been reported as 77% for teenagers aged 13–16 years in 25 European countries [16, 17]. These social network users interact for an average of more than 2.4 hours a day on social media [18]. This increased instant interaction has further increased the use of social media. Beside, social media platforms allows independent sharing, regardless of age, venue, and gender. Thus, information spreads rapidly across a wide area [15, 18]. Additionally, social media platforms ensure simple interaction pathways between people, companies, and scientists without leaving the desk. Therefore, many scientists use social media in their personal or professional lives [19].

The most commonly used platform for the purpose of spreading science is Twitter [20]. Twitter is the most popular microblogging platform nowadays. This platform allows the publication of short messages by its users and enables them to communicate with each other. Evan Williams, Biz Stone, and Jack Dorsey created Twitter in March 2006 and brought into use in July 2006. Twitter has become one of the 10 most visited websites in 2013 and was defined as “the SMS of the Internet” [21, 22]. In 2019, it was reported that there were 330 million monthly and 145 million daily active Twitter users. Nowadays, it was reached 339 million users in 2020 [23]. The number of Twitter users has been increasing daily. Today, Twitter ranks as the world’s second most widely used social network. Twitter users can follow a conversation and discuss a topic using messages named “tweets.” Tweets are constrained to 140 characters of text. Later, this limit was increased to 280 characters. Twitter allows the sharing of photos or videos [24]. Twitter was initially used to share news about the lives of celebrities. Afterward, it reached a broader audience quickly, especially with the participation of famous names and the involvement of political campaigns. On average, approximately 98 thousand tweets are sent every minute on the platform, allowing an excellent interaction. Given the rise in popularity of Twitter, its use is increasing in all parts of society [25]. This widespread use has also caught the attention of the scientific community. The use of Twitter as a tool for the dissemination of academic articles has soon become the focus of attention in the scientific world.

Academic output has increased gradually worldwide. Therefore, eliminating the relevant from the irrelevant has become essential for the scientific world. Thus, an impact scale has been necessitated the published articles [26]. The primary impact of the published article has been measured by its citations [26]. However, article citations have recently become questionable due to negative factors, such as the slow process of identifying truly impactful articles. The long wait time required the emergence of the articles’ importance has led to need for an alternative metric scale [27].

Today, non-traditional metrics, altmetrics, are increasingly used to measure the real-time reach and influence of a scientific article [26, 28]. The term altmetrics was first proposed by Jason Priem in 2010 [29]. Thereafter, it has gained wide use in highlighting previously unknown and unrecognized scholarly impact metrics of studies [26, 30]. The alternative metric scores play a role in complementing traditional metrics or indicators [31]. Many publishers, such as SAGE, Taylor-Francis Group, Elsevier, Nature Publishing Group, and Public Library of Science provide much information to their readers by their altmetrics evaluating system [32]. These altmetrics are calculated by various methods, including “Altmetric” and “Plum Analytics” [33]. All altmetrics that are an alternative to the traditional citation system provide a score for the research output.

These scores have become attractive for researchers [34]. Many scientists believe that these alternative metric scores show the real impact of published articles [35–37]. Many web-based platforms play a significant role in obtaining an altmetric score [11, 31]. With the data obtained from these platforms, a digital score was acquired for academic output [11]. All altmetrics are based on the using social media and other online tools for disseminating scholarly information [38]. The use of social media platforms contributes significantly the spread of shared information in a wider environment. Thus, the sharing of academic output on social media accounts can reach more people faster by eliminating the waiting period in the traditional citation system [39, 40]. They have also allowed the impact of articles to be more immediately determined, contrary to traditional citation metrics [41].

However, some concerns remain that the altmetric score can be manipulated [42, 43]. In particular, the use of automatic bot can affect the altmetric scores of articles [44]. Further, Twitter accounts can affect the results of shared articles. These account holders may be social workers, companies, or politicians, and would have more followers than others [45, 46]. Thus, some researchers suggest the use of “alt‑index” to measure the social visibility of scientific research [6]. Similarly, Haustein et al. argued that social media metrics could not actually be regarded as alternatives to traditional citations; hence, they proposed these metrics as promoters of traditional citations [11]. The authors suggested the use of “Twitter Coupling” to deal with these concerns [38]. Although this may be a solution there is a consensus in many studies that social media usage will increase the impact of academic papers, thus ignoring these concerns [47, 48].

The potential of social media platforms to connect with other fields raises various scientific questions [49]. Therefore, the papers in the social sciences and humanities are more often found on social media platforms [11]. Today, social interaction is so intertwined with media that it is not possible to separate social media from the media sector. Thus, most studies related to social media have been published in the communication sciences. This increased usage of these social networks has led to the research question, “How are altmetrics and citation measures related in communication journals?” Tonia et al. (2016) stated that there were no statistical differences between tweeted and non-tweeted papers regarding their citation numbers [50]. Costas et al. (2015) found only weak correlations in citations suggested by altmetrics and traditional citation analysis [35]. Further, some articles can be more attractive than other on social media platforms. Hence, some researchers have argued that there is a difference between social impact and real impact [11]. However, Thelwall et al. (2013) found that altmetrics was associated with citation counts [51]. Another study stated that there is a positive relationship between social media posts and academic citations [52]. Similarly, Shuai et al. (2012) detected significant correlations between tweets and early citations on 4,606 pre-prints articles [8]. Nevertheless, there is no consensus in the studies that to evaluate between tweeted and non-tweeted papers regarding their citation numbers in communication science, which is the scientific field mostly associated with social media and Twitter. The studies related to social media are encouraged in the field of communication as people present themselves through digitally networked platforms today. Therefore, it was hypothesized that there might be a correlation between Twitter posts and traditional citations the articles in the top ten communication-based journals.

## Materials and methods

### Study design

The present study was designed as a retrospective cross-sectional study. The aim was to examine the effect of Twitter and other social media platforms on academic citations. Therefore, the top ten communication-based journals [9] were evaluated based on the SCImago Journal & Country Rank (cited in 2019). It was used the SCImago Journal & Country Rank search field to select journals and filtered only “communication” journals. The selection criteria were the top ten communication-based journals according to their SCImago Journal Rank indicator. This indicator is a measure of journals’ academic impact and accounts for both the number of citations received by a journal and the importance or prestige of the journals in which the citations [53]. The impact factor of these journals was ≥ 2, and the quartile (Q) index was Q1. All ten journals had similar indexes and similar impact factors. It was assumed that this would reduce the potential for unwanted variation differences in social media activity.

Impact Factor: The impact factor has been defined as an indicator of academic journal. It reflects the year’s average number of citations per paper published during the preceding two years. It is often used as a relative indicator of a journal’s importance in its field. The journals with high impact factors are thought to be more important than those with low impact factors [54].

Q index: The journals’ rank in each specific category is separated into quartiles by the Journal Citation Report and SCImago Journal and Country Rank: Q1, Q2, Q3 and Q4. Q1 comprises the top 25% of journals in the list; Q2, Q3, and Q4 comprise 25% to 50%, 50% to 75%, and 75% to 100% of journals in the list, respectively (https://www.mondragon.edu/en/web/biblioteka/publications-impact-indexes).

These Journals “Political Communication (Q1, IF: 4.339)”, “Journal of Advertising (Q1, IF: 6.302)”, “Journal of Communication (Q1, IF: 4.846)”, Big Data and Society (Q1, IF: 4.577), Applied Linguistics (Q1, IF: 4.286), Communication Methods and Measures (Q1, IF: 5.281), New Media and Society (Q1, IF: 4.577), Human Communication Research (Q1, IF: 3.540), Public Opinion Quarterly (Q1, IF: 2.494), and Digital Journalism (Q1, IF: 4.476) were included in this present study (Table 1).

**Table 1:** The characteristics of journals.

Journals’ Name	5-Years IF	IF (2019)	Q index	Issues (per year)
**Political Communication**	5.068	4.339	Q1	6
**Journal of Advertising**	N/A	6.302	Q1	5
**Journal of Communication**	7.175	4.846	Q1	6
**Big Data and Society**	N/A	4.577	Q1	2
**Applied Linguistics**	4.901	4.286	Q1	6
**Communication Methods and Measures**	N/A	5.281	Q1	4
**New Media and Society**	4.972	4.577	Q1	12
**Human Communication Research**	4.039	3.540	Q1	4
**Public Opinion Quarterly**	3.259	2.494	Q1	5
**Digital Journalism**	N/A	4.476	Q1	10

IF: impact factor; Q index: quartile index; N/A: not available

### Data collection

All issues of these journals in 2018 were reviewed through the journals’ web pages. All published articles in this year were evaluated on the web pages’ archive. The date range was considered based on published issues in the journals. Only original articles (meta-analyses, systematic reviews, original research articles, and research notes) in 2018 were included in this study. Articles such as editorials, book review articles, case reports, letters to the editor, and other non-research correspondences were excluded.

The findings of the articles (title, doi number, article type) were recorded. In January 2021, the title of the article or doi number was searched one by one in Google Scholar (GS) and Web of Science (WoS) Clarivate. The traditional citation numbers (GS and WoS) of these articles were recorded. The tweet number and social media posts of these articles were searched by their metric evaluating system (https://www.altmetric.com/) and recorded. The data were appraised nearly three years after the articles’ publication date. It was thought that this period would allow the impact of published articles (the citations and Twitter mentions) to be fully observed.

## Results

Seven hundred and eleven articles were published in the top ten communication-based journals in 2018. After the exclusion criteria were applied, 572 articles were included for analysis. A total of 570 articles (99.7%) were cited at least once on GS, and 518 articles (90.6%) were cited at least once on WoS. The total cumulative number of citations for all the articles was 21,242 for GS and 5,874 for WoS. The number value of citations ranged from 0 to 868 on GS, and 0–235 on WoS. The median number of citations was 19 citations (interquartile range (IQR): 0–868 citations) for GS, and five citations (IQR: 0–235 citations) for WoS (Table 2).

**Table 2:** The number of citations, mentions of Twitter and other platforms on the articles journal basis.

Journals’ name	Articles (n) (%)	Citations (GS)^≠^	Citations (WoS) ^≠^	Mentions (Twitter) ^≠^	Mentions (Other platforms) ^≠^
**Political Communication**	29 (5.1)	17 (3–171)	2 (0–36)	35 (10–417)	42 (14–342)
**Journal of Advertising**	24 (4.2)	16 (2–203)	1 (0–10)	0 (0–12)	3.5 (0–878)
**Journal of Communication**	57 (10)	15 (2–191)	5 (0–90)	12 (0–301)	39 (0–318)
**Big Data and Society**	39 (6.8)	16 (1–155)	5 (0–42)	33 (10–212)	53 (14–396)
**Applied Linguistics**	39 (6.8)	21 (2–710)	5 (1–235)	2 (0–164)	42 (0–456)
**Communication Methods and Measures**	15 (2.6)	22 (5–156)	2 (0–13)	3 (0–41)	29 (6–303)
**New Media and Society**	245 (42.9)	23 (0–468)	7 (0–168)	8 (0–502)	51 (0–702)
**Human Communication Research**	20 (3.5)	9.5 (2–38)	3.5 (0–19)	2 (0–21)	20 (0–81)
**Public Opinion Quarterly**	30 (5.2)	8.5 (1–121)	3.5 (0–49)	11 (0–374)	18 (0–241)
**Digital Journalism**	74 (12.9)	14.5 (0–868)	3 (0–62)	7 (0–135)	27 (0–1019)
**Total**	572 (100)	19 (0–868)	2 (0–235)	9 (0–502)	39.5 (0–1019)

^≠^ The values were presented as median (minimum-maximum).

In all, 522 articles (91.3%) were posted at least once on Twitter or other platforms. The total number of mentions on all social media platforms was 50,624 items. Overall, the most-used social media platform was Twitter. The majority of articles (n: 500; 87.4%) were mentioned at least once on Twitter, and these articles had cumulatively tweets 13,438 tweet. The median value of Twitter posts was nine tweets (IQR: 0–502 tweet).

The median citation value of articles on GS was 21 citations (IQR: 0–868) for the articles that had been tweeted at least one. However, it was nine (IQR: 0–72) citations for non-tweeted articles. Further, the median WoS citation number was five (IQR: 0–235) for tweeted articles and two (IQR: 0–19) for non-tweeted articles.

The tweeted articles were cited more often than those with no tweets on both platforms (for GS: Mann-Whitney U: 10107, Z: -6.022, p< 0.001; for WoS: Mann-Whitney U: 10547, Z: -5.699, p< 0.001, respectively). This observation was also similar for the other platforms (for GS: Mann-Whitney U: 6493, Z: -5.875, p< 0.001; for WoS: Mann-Whitney U: 6735.5, Z: -5.671, p< 0.001, respectively) (Table 3).

**Table 3:** The articles with exposure social media were related to the higher citation rates.

		Median GS citations	Median WoS citations
**Twitter Mentions**	No	9	2
	Yes	21	5
**P value**		<0.001	<0.001
**Other SoMe Platforms**	No	8	2
	Yes	20	5
**P value**		<0.001	<0.001

GS: Google Scholar; WoS: Web of Science; SoMe: Social Media.

**Fig 1:**
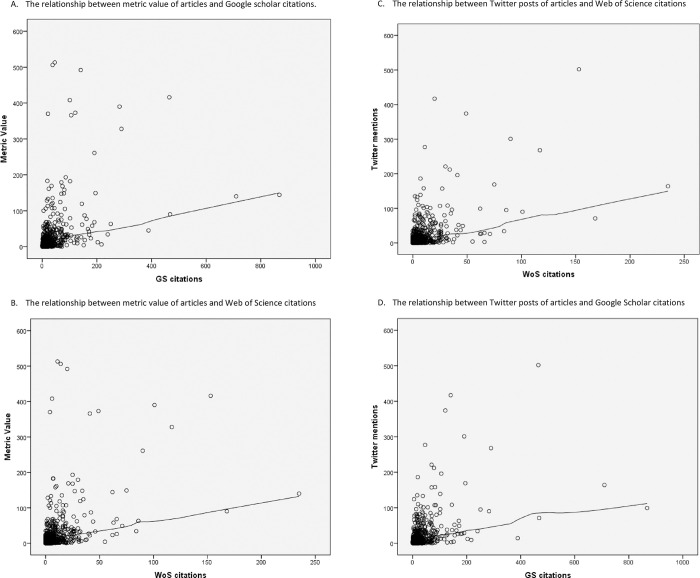
The correlation between citation of articles, and metric value and Twitter posts. A. The relationship between metric value of articles and Google scholar citations. B. The relationship between metric value of articles and Web of Science citations. C. The relationship between Twitter posts of articles and Web of Science citations. D. The relationship between Twitter posts of articles and Google Scholar citations.

It was observed that a significant correlation between the number of Twitter posts and the number of citations in GS (r = 0.44, p<0.001) and in WoS (r = 0.50, P<0.001). Similarly, there was also a positive correlation between the number of mentions on all platforms and the number of citations in GS (r = 0.83, p<0.001) and WoS (r = 0.71, p<0.001) (Fig 1).

## Discussion

Social networks in academia are rapidly improving, and significantly increasing use by the scientific community [55]. Many journals frequently use these tools for advertising and sharing information [56]. Social media platforms have brought another dimension to access the information. With developments in social sharing platforms, there has been a transition to the digital age of accessing information [57]. Thus, these platforms have presented new opportunities for researchers to extend their publications the scientific society [36, 58]. Similarly, this present study found that the articles exposed to social media were cited more than the articles not posted on social media. This study showed that the visibility of articles might be increased by sharing them on social media, which allows the real effects of the articles to emerge more rapidly.

Generally, the use of all networks has a similar effect, but the most used platform for this purpose is Twitter. Twitter allows for the rapid sharing of information within seconds of posting a tweet. Thus, the dissemination rate of tweets increases exponentially [59]. As in other studies, it was found that more use of Twitter than other platforms in this study. Moreover, a positive correlation was observed between the altmetric score, Twitter posts, and citation rate of articles. The articles that were tweeted at least once were cited more than those with no posts on both platforms (for GS, p <0.001; and for WoS, p <0.001). The findings support the conclusion that Twitter activity may reflect the quality of articles or increase their citations. Thus, the measure of social platforms based on tweets should be used to complement the traditional metrics of article citations.

Especially the science of communication is the social sciences field most related to social networks. The traditional publishing continues in the communication sciences, but its adoption of social media-related studies is increasing daily [10, 14]. Therefore, sharing and disseminating the articles in communication journals may significantly increase their citation rates. This present study revealed this relationship and highlighted the impact of using social networks in the academic world. Thus, authors and journals should share all articles using social media tools to increase the impact of the article.

The use of social media platforms in the scientific world will make an important contribution to traditional metric systems. Published articles can be posted on social media to reach more people, disseminate information quickly and increase their impact faster [60]. Further, academic journals’ use of Twitter will promote the journals and increase the citation numbers of the articles [9, 60]. Many journals share all of their articles published on their social media accounts. Some journals only share articles they deem important. However, increasing the visibility of articles on social media platforms could be a tool for reaching more people. Thus, this study shows that Twitter posts could mediate these articles to reach more people.

The results of this study show that articles with exposure to social media had higher citation rates. There was also a positive correlation between exposure to social media and article citations. Therefore, the scientist and journals should develop new projects to increase the usage of social media.

However, the present study has some limitations. First, it analyzed only the number of citations and the number of tweets and did not evaluate their content or the Twitter account holders. Second, this study evaluated only the top 10 journals in the communication science. Third, multiple factors (such as sending articles to the press at the same time, focusing on some specific communication topics, and evaluating popular topics) may play a role in the emergence of the articles’ impact. Posting articles on Twitter is only one contributor to this impact. Further, this study examined the relationship between the number of Twitter posts and the number of citations of articles. However, the cause of the relationship was not analyzed. Therefore, more work is needed to explain potential causes of the relationships between posts and citations of an article. Nevertheless, this study presented significant findings that highlight the importance of using social media in academia.
